# Aberrant miRNAs expressed in HER-2 negative breast cancers patient

**DOI:** 10.1186/s13046-018-0920-2

**Published:** 2018-10-20

**Authors:** Cornelia Braicu, Lajos Raduly, Gabriela Morar-Bolba, Roxana Cojocneanu, Ancuta Jurj, Laura-Ancuta Pop, Valentina Pileczki, Cristina Ciocan, Alin Moldovan, Alexandru Irimie, Alexandru Eniu, Patriciu Achimas-Cadariu, Angelo Paradiso, Ioana Berindan-Neagoe

**Affiliations:** 1 Research Center for Functional Genomics, Biomedicine and Translational Medicine, “Iuliu Hatieganu” University of Medicine and Pharmacy, Cluj-Napoca, Romania; 2 Department of Senology, The Oncology Institute “Prof. Dr. Ion Chiricuta”, Cluj-Napoca, Romania; 3 Department of Analytical Chemistry, Faculty of Pharmacy, “Iuliu Hatieganu” University of Medicine and Pharmacy, Cluj-Napoca, Romania; 4 MedFuture Research Center for Advanced Medicine, “Iuliu Hatieganu” University of Medicine and Pharmacy, Cluj-Napoca, Romania; 5 Department of Surgery, The Oncology Institute “Prof. Dr. Ion Chiricuta”, Cluj-Napoca, Romania; 6 Department of Surgical Oncology and Gynecological Oncology, University of Medicine and Pharmacy Iuliu Hatieganu, Cluj-Napoca, Romania; 7 National Cancer Research Centre, Istituto Tumori G Paolo II, Bari, Italy; 8 Department of Functional Genomics and Experimental Pathology, The Oncology Institute “Prof. Dr. Ion Chiricuta”, Cluj-Napoca, Romania

**Keywords:** Triple negative breast cancer, Double positive breast cancer, Plasma miRNA

## Abstract

**Background:**

Breast cancer is a highly heterogeneous pathology, exhibiting a number of subtypes commonly associated with a poor outcome. Due to their high stability, microRNAs are often regarded as non-invasive cancer biomarkers, having an expression pattern specific for their ‘cell of origin’.

**Method:**

Triple negative breast cancer (TNBC: ER-, PR-, Her-2-) and double positive breast cancer (DPBC: ER+, PR+, Her-2) miRNA expression patterns were obtained by analysis of the TCGA (*The Cancer Genome Atlas) data, followed by PCR-array analysis* on plasma samples from 20 TNBC patients, 14 DPBC patients and 11 controls.

**Results:**

Three downregulated and nine upregulated miRNAs were obtained from the TNBC analysis. Five overexpressed miRNAs were identified in the DPBC group. Four of the dysregulated miRNAs (miR-10a, miR-125b, miR-210 and miR-489) were common for both groups. The cluster miR-17-92 (miR-17, miR-20a, miR-20b, and miR-93), along with miR-130, miR-22 and miR-29a/c, were found to differentiate between TNBC and DPBC. A panel of five transcripts (miR-10a, miR-125, miR-193b, miR-200b and miR-489) was validated in a new set of plasma samples. The overlapping of TCGA and plasma profiling data revealed miR-200b, miR-200c, miR-210 and miR-29c as common signature. MiR-200b was validated on additional normal and tumor tissue samples. The expression level of this transcript from the TCGA data was correlated with lung and bone metastatic genes.

**Conclusion:**

The miR-200b presents a great potential for the future advancements in the diagnostic/prognostic and therapeutic approach of TNBC, along with other coding or non-coding transcripts. However, this needs to be further integrated in a regulatory network that acts in conjunction with other markers that affect the patients’ prognosis or response to therapy.

**Electronic supplementary material:**

The online version of this article (10.1186/s13046-018-0920-2) contains supplementary material, which is available to authorized users.

## Background

Breast cancer (BC) is the most common malignant pathology affecting women worldwide [1–3]. As BC accounts for an increasing number of deaths each year, efforts are being made to develop more efficient methods for early diagnosis, stratification and prediction of therapy response. The complexity of this disease comes from the diversity of environmental factors along with various inhered or acquired genomic, transcriptomic or proteomic alterations [4]. In general, BC is classified based on the expression levels of estrogen receptor (ER), progesterone receptor (PR) and human epidermal growth factor receptor 2 protein (HER-2). Triple negative breast cancer (TNBC) represents about 15–20% of BC cases [5, 6], and is characterized by the absence of ER, PR and Her-2 proteins [2, 4, 5]. This BC subtype poses major clinical challenges due to the lack of specific diagnostic/prognostic biomarkers and the failure of standard therapy to provide a targeted effect [2, 6–8].

MicroRNAs (miRNAs) are short noncoding RNAs of about 19–25 nucleotides in length [9–11]. MiRNA profiling studies have identified specific miRNA signatures in a wide range of cancer types [12–14]. These transcripts can either be overexpressed (oncomiRs) or underexpressed (tumor suppressor miRs) [4, 11, 15, 16]. These alterations are specific for each malignancy, including various BC subtypes [4, 5, 7, 9, 15–18]. Thereby, circulating miRNAs are potential biomarkers in the case of numerous diseases [19], such as BC [15, 20–22]. The studies undertaken to prove the causative effect of miRNA first perform a general profiling of clinical samples, then are followed by controlled experiments [22–26]. Still many questions remain regarding the exact mechanisms, biological functions, and clinical implication of miRNAs in the BC subtypes [11, 17, 21].

**The Cancer Genome Atlas (TCGA)** is a large database of sequencing results generated from studies involving genome analysis in a rigorous and consistent manner [27]. This allowed us to perform a direct comparison between the TCGA data and the results from our PCR-array plasma profiling study of TNBC and DPBC. We evaluated a panel of miRNAs related to BC and we identified the most specific miRNAs for TNBC and DPBC. The validation was done in a new independent patient cohort with the help of qRT-PCR technology. Furthermore, by overlapping the miRNA patterns, we identified either common or specific miRNA signatures for the two selected subtypes of Her-2 negative BC. Based on the expression level of the transcripts, miRNAs survival curves were generated. The results revealed the prognostic potential of some miRNAs, as well as their interdependence with some metastasis related genes.

## Methods

### TCGA miRNA expression pattern evaluation

We downloaded level 3 TCGA data from the University of California Santa Cruz cancer genomics data portal in the form of data matrices documenting patterns of miRNA expression for 112 TNBC tissue samples, 358 DPBC tissue samples, and 44 normal tissues (Table 1). Differential expression analysis was performed using the GeneSpring GX software from Agilent Technologies. The volcano plot module was applied, using a fold change > 1.5 and a *p*-value of < 0.05. An additional validation step was performed for miR-200b in normal (*n* = 19), DPBC (*n* = 47) and TNBC (*n* = 21) tissues (Table 2), in order to sustain the plasma expression profiling and the TCGA data, displayed as Pirate Plot generated in R programme.

**Table 1:** TGGA patient cohort characteristics

Demographics	TNBC (*n* = 112)	DPBC (*n* = 358)
Sex		
Males	0	3
Females	112	355
Age		
Median, Range	54, 29–90	58, 28–90
Median, Range ♂	–	68, 44–84
Median, Range ♀	54, 29–90	58, 28–90
Menopausal status		
Pre-menopausal	30	89
Peri-menopausal	5	16
Post-menopausal	68	225
Unknown, N/A	9	28
TNM		
T1	27	110
T2	70	189
T3	11	48
T4	4	10
Tx	–	1
N0	72	168
N1	25	121
N2	11	39
N3	4	25
Nx	–	5
M0	95	308
Mx	17	50
Turmor grade		
I	20	72
II	70	195
III	18	82
IV	1	3
X / unknown	3	6

**Table 2:** Clinical characteristic of patients with TNBC and DNBC patient cohort for PCR-array screening profile and plasma qRT-PCR validation lot

No	TNM stage	Age
TNBC		
1	T4bN1 M0	56
2	T2N0M0	59
3	T4bN2Mx	40
4	T2N0M0	52
5	T2 N1 M0	46
6	T2N0M0	53
7	T2 N1 M0	56
8	T3 N1 M0	46
9	T4bN1 M0	57
10	T3 N1 M0	50
11	T4bN2Mx	57
12	T4bN2M0	55
13	T2 N1 M0	35
14	T4cN2Mx	59
15	T2 N1 M0	48
16	T4bN1 M0	50
17	T2 N1 M0	51
18	T3 N1 M0	59
19	T3 N1 M0	45
20	T4bN1 M0	56
21	T3 N1 M0	53
DPBC		
1	T2N1aMx	59
2	T2 N1 M0	69
3	T3N1Mx	60
4	T2N0Mx	39
5	T4bN3aMx	73
6	T2N0M0	49
7	T2N0Mx	42
8	T3N1Mx	58
9	T2 N1 M0	41
10	T1N0Mx	67
11	T4bN1 M0	66
12	T3N1Mx	52
13	T2N2aMx	57
14	T4bN1 M0	52
15	T1N0Mx	42
16	T4bN1 M0	38
17	T2N1Mx	62
18	T2N0M0	46
19	T3N0M0	57
20	T2N2aMx	48
21	T2 N1 M0	64
22	T3N1Mx	63
23	T2N0M0	62
24	T4N3bMx.	70
25	T2N0M0	62
26	T3N1aMx	66
27	T1N0M0	69
28	T3N1Mx	45
29	T2 N1 M0	44
30	T3N1Mx	36
31	T2N0M0	42
32	T3N0Mx	47
33	T2N1Mx	47
34	T2 N1 M0	41
35	T4N2Mx	51
36	T2 N1 M0	44
37	T4N2Mx	45
38	T3N0Mx	37
39	T4N2Mx	73
40	T3N1Mx	40
41	T4N2Mx	49
42	T4N1Mx	56
43	T3N3Mx.	80
44	T3N3Mx.	49
45	T4N2Mx	59
46	T3N0Mx	49
47	T2 N1 M0	59

### Survival analysis for the TCGA patients

We extracted the patient survival data from the TCGA clinical information file. In the case of miR-200b, miR-200c, miR-210, and miR-29, the survival was estimated in days from the date of diagnosis until date of last contact. Survival analysis was performed by using Kaplan Meier curves, in the GraphPad Prism program. In addition, we assessed the correlation of miR-200b to the most relevant metastatic markers, as described in literature [28, 29].

### Sampling procedures

The sampling for all biological specimens was done after we received the approval from the Oncology Institute “Prof. Dr. Ion Chiricuta” Ethics Committee and the informed consent form signed by the patient. The patients were diagnosed at the Oncology Institute “Prof. Dr. Ion Chiricuta” in Cluj-Napoca, Romania. The clinical characteristics of patients are presented in Table 3. The blood samples were collected from patients with TNBC or DPBC prior to treatment, between November 2010 and August 2013. In addition, blood samples from eight healthy female controls, free of any chronic diseases, were obtained in the second half of 2013. Sampling for all biological specimens was performed according to Romania’s laws and accompanied by an informed consent signed by every donor. The peripheral blood samples were collected in 3 ml tubes with EDTA for plasma isolation, and prepared by centrifuging the blood at 3000× rpm for five minutes. The plasma supernatant was carefully removed, placed in 2 ml Eppendorf tubes, and stored at − 80 °C. The qRT-PCR for miRNA-39 was used as quality control for extraction efficiency and as an indicator of miRNA recovery rate from plasma.

**Table 3:** Clinical characteristic of patients with TNBC and DNBC patient cohort for PCR-array screening profile and plasma qRT-PCR validation lot

No	TNM stage	Age
PCR-array plasma		
TNBC		
1	T4bN3M0	58
2	T2 N1 M0	47
3	T3N2M0	59
4	T4bN1 M0	51
5	T3 N1 M0	45
6	T4bN2M0	51
7	T2 N1 M0	51
8	T2 N1 M0	56
9	T4bN2M0	43
10	T2 N1 M0	35
11	T2N2Mx	53
12	T2 N1 M0	40
13	T4cN2Mx	59
14	T4bN2M0	55
15	T1N0M0	48
16	T1 N1 M0	56
17	T2N2Mo	54
18	T4bN2Mx	40
19	T2N0M0	52
20	cT2 N1 M0	59
DPBC		
1	T2N2M0	54
2	T2N2M0	52
3	T4bN2Mx	72
4	T4bN2M0	62
5	T3 N1 M0	62
6	T2N1Mx	52
7	T2N1Mo	51
8	T2 N1 M0	45
9	T3N0Mx	43
10	T3 N1 M0	57
11	T2N0M0	48
12	T1N0M0	56
13	T4aN0M0	53
14	T2N0M0	62
qRT-PCR plasma		
TNBC		
1	T4bN1 M0	56
2	T2N0M0	59
3	T2N3cM0	58
4	T2 N1 M0	57
5	T2 N1 M0	46
6	T2N0M0	53
7	cT2N2M0	59
8	T4bN2M0	73
9	cT1N0M0	70
10	T2N2Mx	49
11	cT4bN2M0	61
12	cT4bN2Mx	57
13	cT2N1Mx	74
14	T2N0M0	53
15	T2N0M0	34
16	T2N1cM0	62
17	T4bN2M0	46
18	T1 N1 M0	38
19	T3 N1 M0	40
20	T2 N1 M0	35
21	T2N0M0	36
22	T2N0M0	37
23	T2N0M0	34
24	T2N0M0	36
DPBC		
1	T2 N1 M0	54
2	T2NoMo	59
3	T2 N1 M0	52
4	T2N0M0	46
5	T4bN2Mx	60
6	T3N1Mx	63
7	T2N0M0	67
8	T4bN2M0	53
9	T3N1Mx	43
10	T2N0M0	51
11	T2 N1 M0	64
12	T2N1Mo	57
13	T4bN2M0	45
14	T3N0Mx	69
15	T2N1Mx	52
16	T2 N1 M0	44
17	T2 N1 M0	55
18	T2 N1 M0	62
19	T1 N1 M0	49
20	T3N1Mx	40
21	T3N0Mx	45
22	T3N1Mx	60
23	T4N2M0	63
24	T4 N1 M0	50
25	T2N1Mx	65
26	T2 N1 M0	60
27	T2NoMo	44
28	T4bN2Mo	47

### miRNA isolation from plasma samples

Before use, plasma samples were thawed for five minutes on ice. Total circulating miRNAs were isolated from a 200 μl plasma aliquot using a commercially available column-based assay, according to the manufacturer’s instructions (Qiagen miRNeasy Serum/Plasma Kit). Spike-in control, containing lyophilized *C. elegans* miR-39 miRNA mimic was added to each sample, used as a PCR normalization control. In the final elution stage, 14 μl of RNase-free water were added to the membrane of the MinElute spin column. This was incubated for 1 min at room temperature and centrifuged at 1200 g for another minute. The isolated miRNA samples were stored at − 20 °C before processing.

### PCR array analysis

To generate the cDNA, we used the miScript HiSpec Buffer and 2 μl of total RNA. The 20 μl amplification mixture was incubated at 37 °C for 60 min, then at 95 °C for 5 min. The cDNA was then diluted and mixed with the miScript miRNA PCR array kit, containing specific miRNA primers and QuantiTect SYBR Green PCR Master Mix. For the PCR array analysis, we worked with the 96-well Human Breast Cancer miScript miRNA PCR Array (SABiosciences), containing replicates for miRNA reverse transcription control assay (miRTC) and a positive PCR control (PPC). The plate contains probes for 84 miRNAs whose expression is known or expected to be altered in breast cancer. The miScript SYBR Green PCR Kit was used following the manufacturer protocol, with one exception: only half of the cDNA volume was used and therefore 50 μl of RNase free water was added at the total volume of the reaction mixture. For the PCR-array determination, the Roche LightCycler480 instrument was used, following the cycling conditions indicated by the producer.

The miRNA PCR-array data analysis is displayed as fold-change mean for TNBC group, compared with the healthy female controls. For the interpretation of data, we used a web analysis tool provided by Qiagen, USA (https://www.qiagen.com/us/shop/genes-and-pathways/data-analysis-center-overview-page/), based on the ΔΔc_t_ method for the calculation of relative miRNA expression. The normalization was done with the help of the average Ct value and the reference expression of cel-miR-39, SNORD68, SNORD95, SNORD96A, RUN6–2.

### qRT-PCR data validation

To perform data validation, samples from 28 healthy controls, 24 TNBC and 24 DPBC were analyzed. For the cDNA protocol, we took a total of 50 ng of isolated RNA and mixed it with the Taqman microRNA Reverse Transcription Kit (Cat. No. 4366596, Life Technologies) in a reaction volume of 7.5 μl. Then the following cycling parameters were utilized: 16 °C for 30 min, 42 °C for 30 min, 85 °C for 5 min. The qRT-PCR reaction was performed on the ViiA7 instrument (Applied Bio systems) by using 5 μl of SsoFast Supermix (Biorad cat no. 172–5230), 4.5 μl of 5X diluted cDNA and 0.5 μl of TaqMan Primer. The evaluated miRNAs were: miR-10a, miR-125, miR-193b, miR-200b and miR-489. For data normalization of miRNA expression levels, U6 was used. The same protocol was used for the miR-200b tissue validation. When normalizing this data set, we used U6, RNU48 and miR-16. The qRT-PCR cycle was set at: 98 °C for 3 min, 40 cycles of 95 °C for 15 s, 60 °C for 30 s. The data were analysed by applying the ΔΔCt method and presented as Pirate Plot using R.

## Results

### Evaluation of altered tissue miRNA pattern in TNBC and DPBC using TCGA data

**Fig. 1:**
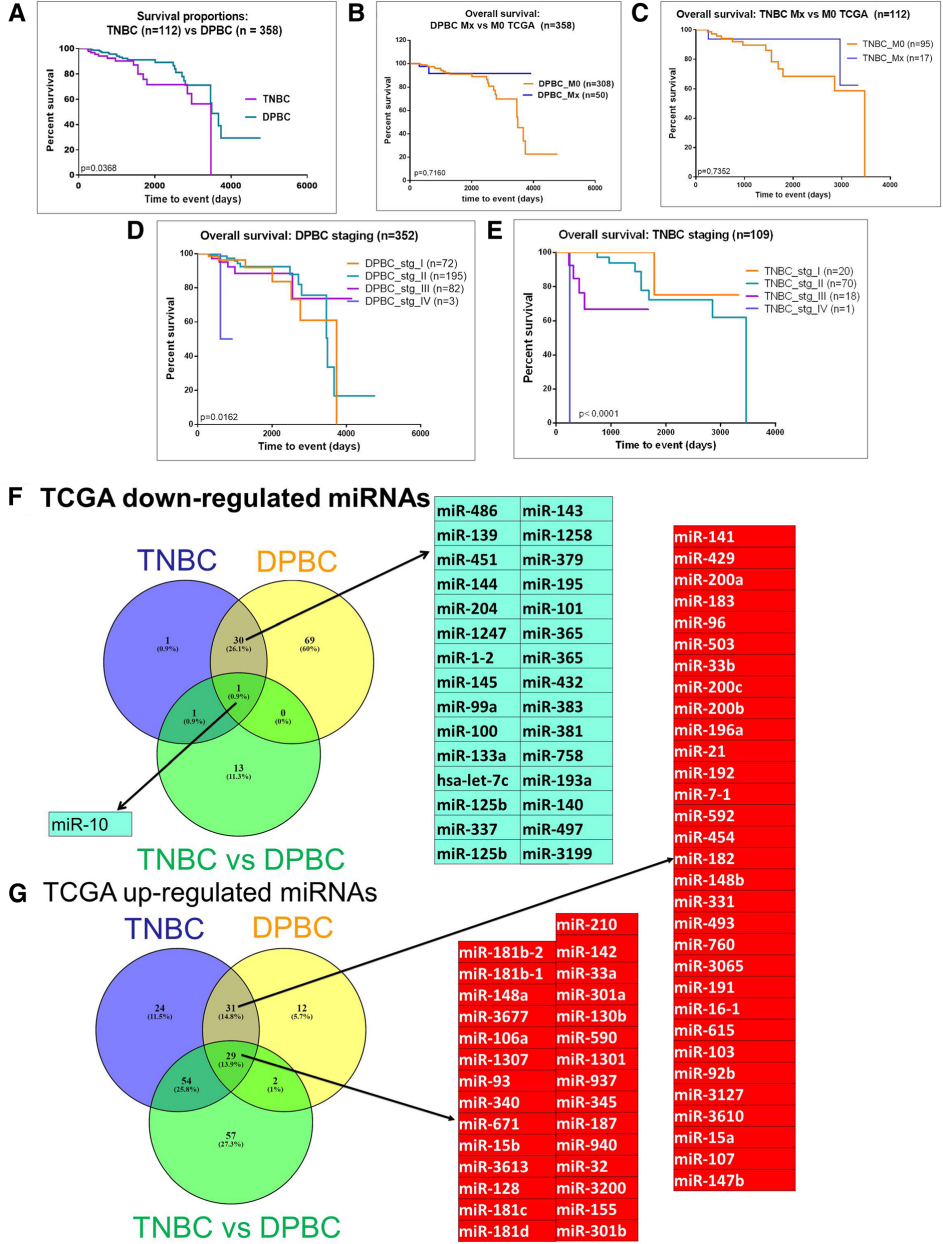
Evaluation of altered tissue miRNA pattern based on TCGA data. (**a**) Kaplan-Meier analysis of 112 TNBC and 358 DPBC patients from TCGA database; (**b**) the analysis of the overall survival rate in TNBC cases with metastases (*n* = 17) versus those without metastases (95); (**c**) analysis of the overall survival rate in TNBC cases with metastases (*n* = 17) versus those without metastases (*n* = 95), (**d**) and (**e**) cumulative disease-free survival separated on staging for TNBC, respectively for DPBC, (**f**) and (**g**) Venn diagram, depicting the overlap between the miRNA detected as overexpressed or downregulated in the TNBC versus normal tissue, DPBC versus normal tissue, respectively TNBC versus DPBC patient cohort, based on the TCGA data

The overall survival rates for the TNBC and DPBC patient cohorts are presented in Fig. 1a. TNBC had a lower survival rate than DPBC. No significant difference was found among the patients with metastases versus those without metastases (Fig. 1b-c). When the cases were separated based on the disease stage, we found that there was a statistically significant difference in only one case, namely stage IV TNBC. Therefore, we did not take it into consideration for further analysis. For the rest of the stages, the differences were not statistically significant (Fig. 1d-e).

To delineate specific miRNAs for pathological differentiation like those associated with TNBC and DPBC**,** we did a profiling analysis using the miRNA expression values from the TCGA database (level 3 accessibility). The extracted data came from 112 TNBC tissue samples, 358 DPBC tissue samples and 44 normal tissue samples. We used a cut-off value of 1.5 for the fold change and of 0.05 for the *p*-value. The comparison between tumors and normal tissue identified 33 down-regulated miRNAs and 138 up-regulated miRNAs in TNBC. Specifically for DPBC, 100 miRNAs were underexpressed and 74 miRNAs were overexpressed. In the case of TNBC versus DPBC, we found 15 downregulated and 142 overexpressed transcripts. Further details on the TGCA data analysis are found in Additional files 1, 2, 3: Table S1-S3. Based on this data, we also constructed heatmaps for the analyzed groups. The aforementioned heatmaps are as follows: Additional file 4: Figure S1 for TNBC vs. normal tissue; Additional file 5: Figure S2 for DPBC vs. normal tissue, and Additional file 6: Figure S3 for TNBC vs. DPBC. A summary of the above mentioned data is presented in Fig. 1f-g, which consists of a list with the miRNA expression profiles common for both BC subtypes and an intersection profile for the up- or down-regulated miRNAs in the two Her-2- BCs. These results illustrate the miRNAs pattern specific for each BC subtype.

Plasma miRNA profiling in TNBC and DPBC. Validation of the most relevant altered transcripts.

**Fig. 2:**
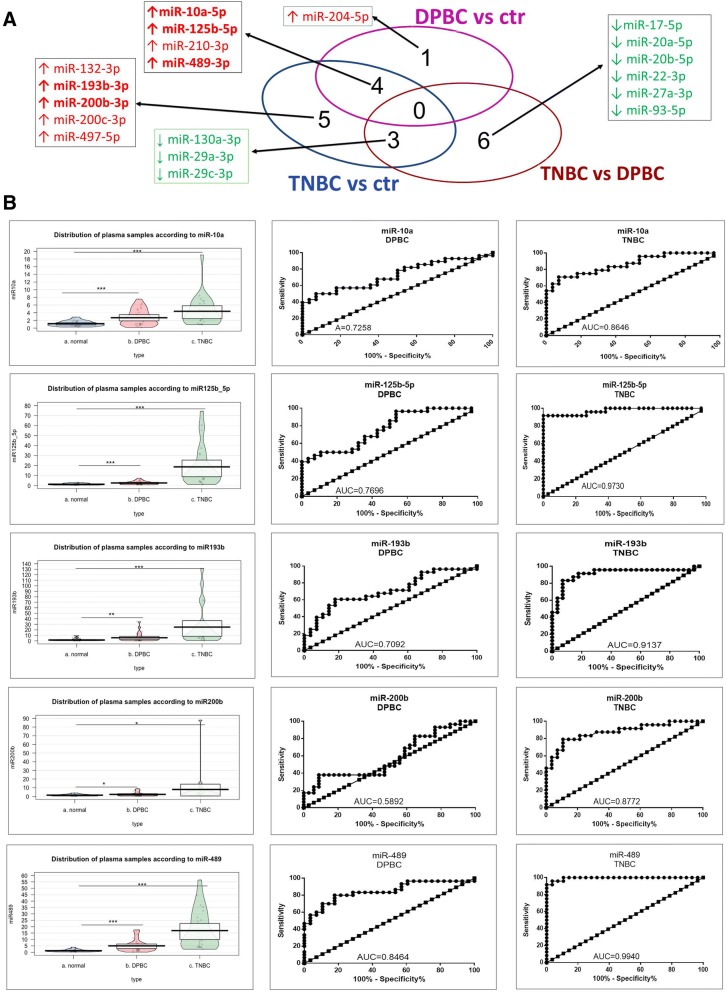
Evaluation of altered plasma miRNA pattern. (**a**) Venn diagram depicting the overlap between the miRNA detected by PCR-array analysis of selected groups, those displayed in bold are the transcripts selected for validation in a new patient cohort; (**b**) Expression levels displayed as Pirate Plot for miR-10a, miR-125, miR-193b, miR-200b and miR-489 in the plasma of 28 healthy controls, 28 DPBC patients and 24 TNBC patients. The ROC curves were used to compare the capacity of miRNA to distinguish between the TNBC/DPBC patients and the healthy controls. This was done only for the validated transcript

The miRNA profiling study for plasma samples was conducted on a total of 45 patients. The immunohistochemistry (IHC) analysis had previously revealed that 20 cases had TNBC and 14 cases had DPBC. A PCR-array study was performed, based on the SABiosciences technology. The panel contained 84 miRNAs recognized as being involved in BC development and progression. The data was normalized with the help of cel-miR-39, SNORD68, SNORD95, SNORD96A, RUN6–2. The miRNAs with a > 1.5-fold expression difference and *p*-value of < 0.05 were further taken into consideration. Table 4 presents the differentially expressed miRNAs organized as follows: TNBC vs. Control; DPBC vs. control, and TNBC vs. DPBC. In addition, the heatmap for these results can be seen in Additional file 7: Figure S4. Fig. 2a is a Venn diagram summarizing the commonly altered miRNA transcripts in the analyzed groups. In TNBC versus control comparison, twelve miRNAs were differentially expressed (respectively, nine up- regulated and three down-regulated). Five miRNAs were found to be overexpressed characteristically in the DPBC group.

**Table 4:** Plasma microRNAs differentially expressed for selected groups (fold change ≤ − 1.5 or ≥ 1.5, *p*-value < 0.05)

TNBC vs ctrl			
A12	miR-10a-5p	4.6091	0.000083
B02	miR-125b-5p	2.5615	0.02088
B08	miR-132-3p	3.6063	0.004549
D02	miR-193b-3p	7.5449	0.000075
D09	miR-200b-3p	4.7585	0.003862
D10	miR-200c-3p	4.2398	0.013366
E07	miR-210-3p	4.1482	0.001688
G03	miR-489-3p	6.7318	0.006437
G05	miR-497-5p	7.4127	0.000067
B06	miR-130a-3p	−2.1947	0.044167
F06	miR-29a-3p	−1.8771	0.039686
F08	miR-29c-3p	−1.9793	0.04623
DPBC vs ctrl			
A12	miR-10a-5p	3.3504	0.000725
B02	miR-125b-5p	2.2752	0.024677
E01	miR-204-5p	4.0558	0.000389
E07	miR-210-3p	3.1667	0.018048
G03	miR-489-3p	4.9291	0.000318
TNBC vs DPBC			
B06	miR-130a-3p	−2.2079	0.043226
C06	miR-17-5p	−2.0127	0.02068
E04	miR-20a-5p	−1.9896	0.032844
E05	miR-20b-5p	−2.0312	0.020571
E10	miR-22-3p	−3.5484	0.009385
F04	miR-27a-3p	−1.9117	0.047502
F06	miR-29a-3p	−2.1888	0.009944
F08	miR-29c-3p	−2.1302	0.018797
G10	miR-93-5p	−2.1871	0.014935

The expression levels of the five most altered miRNAs (miR-10a, miR-125, miR-193b, miR-200b and miR-489) were validated with the help of qRT-PCR in a new patient cohort of 24 TNBC patients, 28 DPBC patients, and 28 healthy individuals. The miRNA expression levels were normalized with U6. All samples were evaluated in duplicate and the geometric mean values were used for data analysis. This resulted in all of the five transcripts being significantly overexpressed in both DPBC and TNBC (data displayed as Pirate Plot in Fig. 2b), thereby validating the PCR-array data. The ROC (Receiver operating characteristic) was used to test the specificity and sensitivity of miRNA relative expression level in both groups (TNBC and DPBC), as well as to distinguish between plasma samples from BC patients vs. healthy controls. The ROC curve analysis showed that miR-125b, miR-193b, miR-200b, and miR-489 could serve as potential biomarkers for discriminating TNBC patients from healthy controls, with AUC (area under the curve) calculated based on ROC curves being 0.9730, 0.9137, 0.8772 and 0.9940.

### Venn diagram analysis of altered miRNA in plasma and the tissue subgroups. Survial rate for the relevant common transctripts

**Fig. 3:**
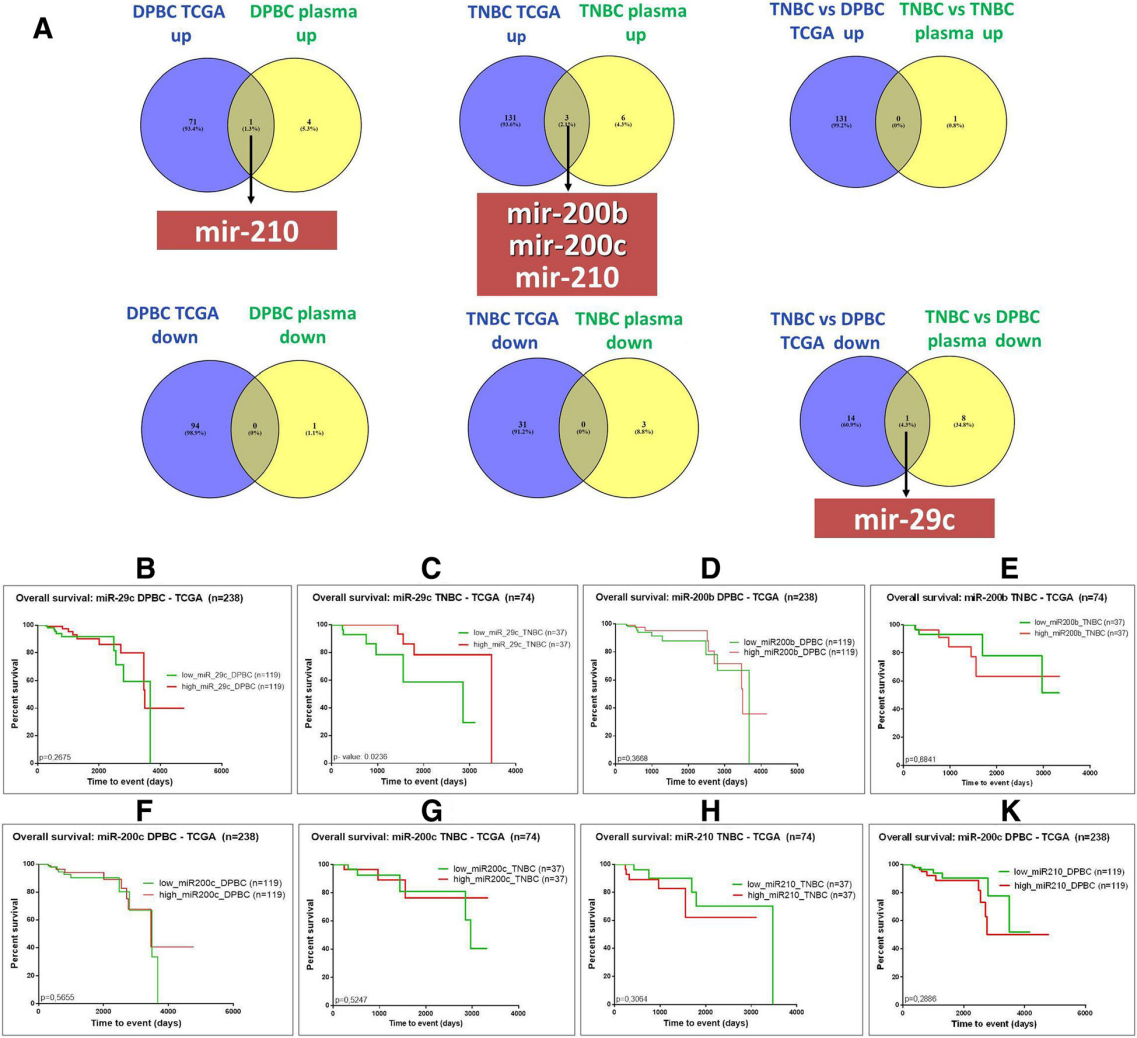
Survial rate for relevant transctrips. (**a**) Venny diagram depicting the overlap of the tissue from TCGA evaluation and plasma data, showing the common miRNA with an altered expression level; (**b**-**k**) overall survival for high and low expression levels of miR-29, miR-210, miR-200b and miR-200c in the case of TNBC and DPBC groups

The altered miRNA expression in plasma and tissue were graphed in the Venn diagram. This was done in order to identify the transcripts with the highest potential of becoming diagnostic/prognostic biomarkers. Fig. 3a shows a list of the miRNA expression profiles common in all groups as well as the overlap between tissue and plasma data. This separates the miRNAs that are specific for each BC subtype, taking into consideration the same altered expression both in tumor and in plasma. In the case of TNBC, the miRNAs common to the other groups were: miR-200b, miR-200c and miR-210. More exclusively, miR-210 was found to be specific for TNBC while miR-29c can be used to differentiate between TNBC and DPBC.

The clinical relevance of miR-29c, miR-200b, miR-200c and miR-210 was also analyzed by means of Kaplan–Meier survival plots. There was no statistically significant (Fig. 3 B-K) difference between patients with low vs. high expression of these miRNAs. This further proves the complex biology of cancer, which cannot be limited to a single biomarker.

### Plasma miRNA biological networks with clinical implication

The role of miRNAs in BC pathogenesis is strongly influenced by the complex interactions miRNAs establish with their targeted mRNAs and other miRNAs. Therefore, we decided to construct a miRNA-mRNA interaction network in the Ingenuity Pathway Analysis (IPA) Software. This was done for both BC subtypes. At the same time, the network revealed the altered pathways specific either for TNBC or for DPBC. The main biological functions affected by the targeted genes were related to cellular development, cell growth and proliferation or invasion (Table 5). In addition, several miRNA were proven to target epithelial to mesenchymal transition (EMT), specifically for the TNBC group.

**Table 5:** miRNAs found to be involved in cellular bio functions cancer

	TNBC		DPBC		TNBC versus DPBC	
	*p*-value	Molecules	*p*-value	Molecules	*p*-value	Molecules
Cancer	1.33E-15 - 4.83E − 02	10	3.31E-10 - 3.95E − 02	5	4.16E − 09 - 4.42E − 02	5
Cellular Development	4.98E − 07 - 4.44E − 02	8	1.70E − 04 - 4.84E − 02	4	9.03E − 06 - 4.72E − 02	3
Cellular Growth and Proliferation	4.98E − 07 - 4.44E − 02	8	1.54E − 03 - 3.95E − 02	4	9.03E − 06 - 4.72E − 02	3
Cell Death and Survival	1.02E − 04 - 4.00E − 02	7	1.80E − 03 - 8.20E − 03	3	–	–
Cellular Movement	1.42E − 05 - 4.34E − 02	6	4.88E − 03 - 3.00E − 02	2	–	–
Cell Cycle	–	–	**–**	–	5.14E − 04 - 1.69E-2	2
Cell morphology	–	–	**–**	–	5.14E − 04 - 1.23E − 02	3

**Fig. 4:**
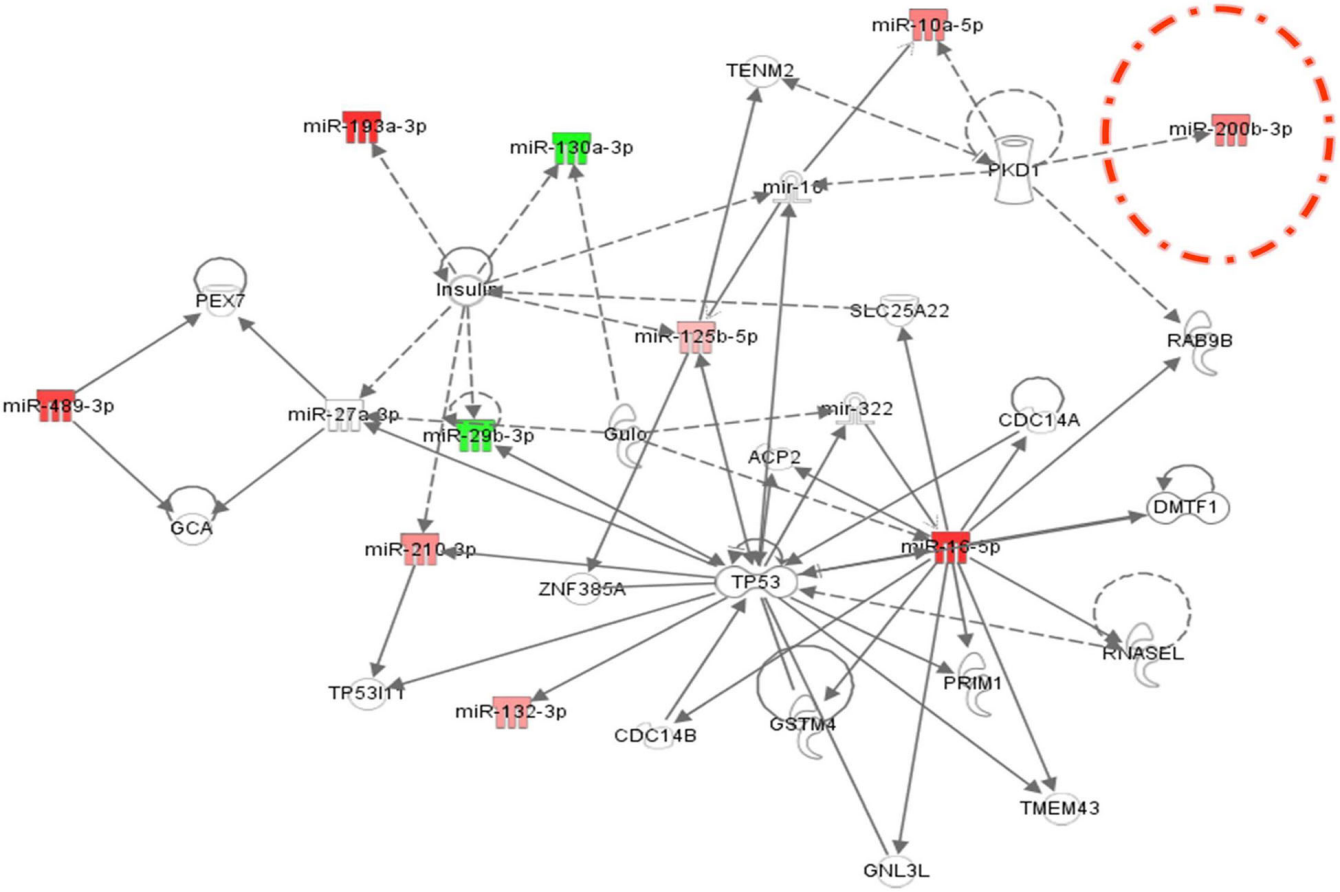
Network connection between miRNAs found to be involved in regulating the expression of genes related to TNBC (upregulated miRNAs are displayed in red and downregulated miRNAs are displayed in green). The network was generated by using IPA (Ingenuity Pathway Analysis)

Using IPA, we were able to generate a miRNA-mRNA interaction network for the miRNAs with altered expression in the plasma from the two HER2- BC patient groups. Fig. 4 represents the miRNA-mRNA interaction network for the TNBC group. The miRNAs that have modified expression values have been color-coded: red for overexpression and green for underexpression. These miRNAs are interconnected with genes involved in apoptosis, cell cycle progression, carcinogenesis and invasion**.** Therefore, the analysis of biological networks revealed a common miRNA-targeted signature, found to be involved in regulating most of same genes as in the tumor tissue. The TP53 gene is central to this network as it establishes a number of connections with the analyzed miRNAs.

### Validation of miR-200b expression level in TNBC and DPBC tissues

**Fig. 5:**
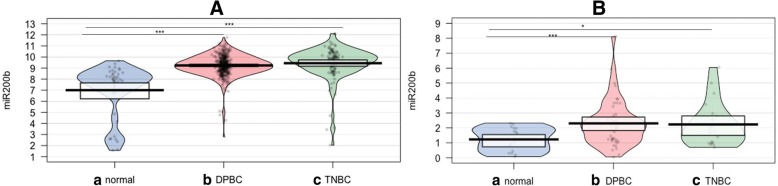
Validation of miR-200b expression level in TNBC and DPBC tissues**.** (**a**) miR-200b expression level in TCGA patient cohort displayed as Pirate Plot, comprising DPBC (*n* = 352) and TNBC (*n* = 109) tissues, reported to normal tissue (*n* = 44) (**b**) miR-200b expression level on normal (*n* = 19), DPBC (*n* = 47) and TNBC (*n* = 21) tissues

Fig. 5a illustrates the higher expression level of miR-200b in the TNBC tissue (*n* = 109) and DPBC (*n* = 358) tissue compared to the normal tissue (*n* = 44). A second validation step was done for the TNBC (*n* = 21) tissue samples and DPBC (*n* = 47) tissue samples, each respectively compared to normal tissue samples (*n* = 19). This analysis further confirmed the TCGA data, demonstrating that miR-200b is overexpressed in both BC subtypes. Moreover, miR-200b was found to be up-regulated in the plasma from both TNBC and DPBC patients, further validating its potential use as a BC biomarker. The miR-200b targeted genes are presented in Fig. 5b. The analysis was done with the online software TargetScan http://www.targetscan.org/vert_72/. The gene list was then integrated in String https://string-db.org, in order to assess the connection network established between the targeted genes.

### Correlation of miR-200b expression levels with metastatic gene markers

Metastasis formation represents a crucial step in the progression of all cancer types. In BC, each site of metastasis is predicted by set of makers. Therefore, we investigated the relationship between miR-200b and various metastasis associated genes in the DPBC and TNBC tumors, being selected the specific genes to the brain (BRCA2 and PARP1), to the lungs (TFF1 and RARA), to the liver (CDH2 and ERCC2) and to the bone (MTA1, KPNA2, BMP2, BMP4, VIM, CD44, PTX3, TNFSF11, CTNNB1, NFKB1, VDR). The TCGA data containing the expression levels of mRNA and miRNA was retrieved in the form of separate data matrices from the same online source (UCSC data portal).

**Fig. 6:**
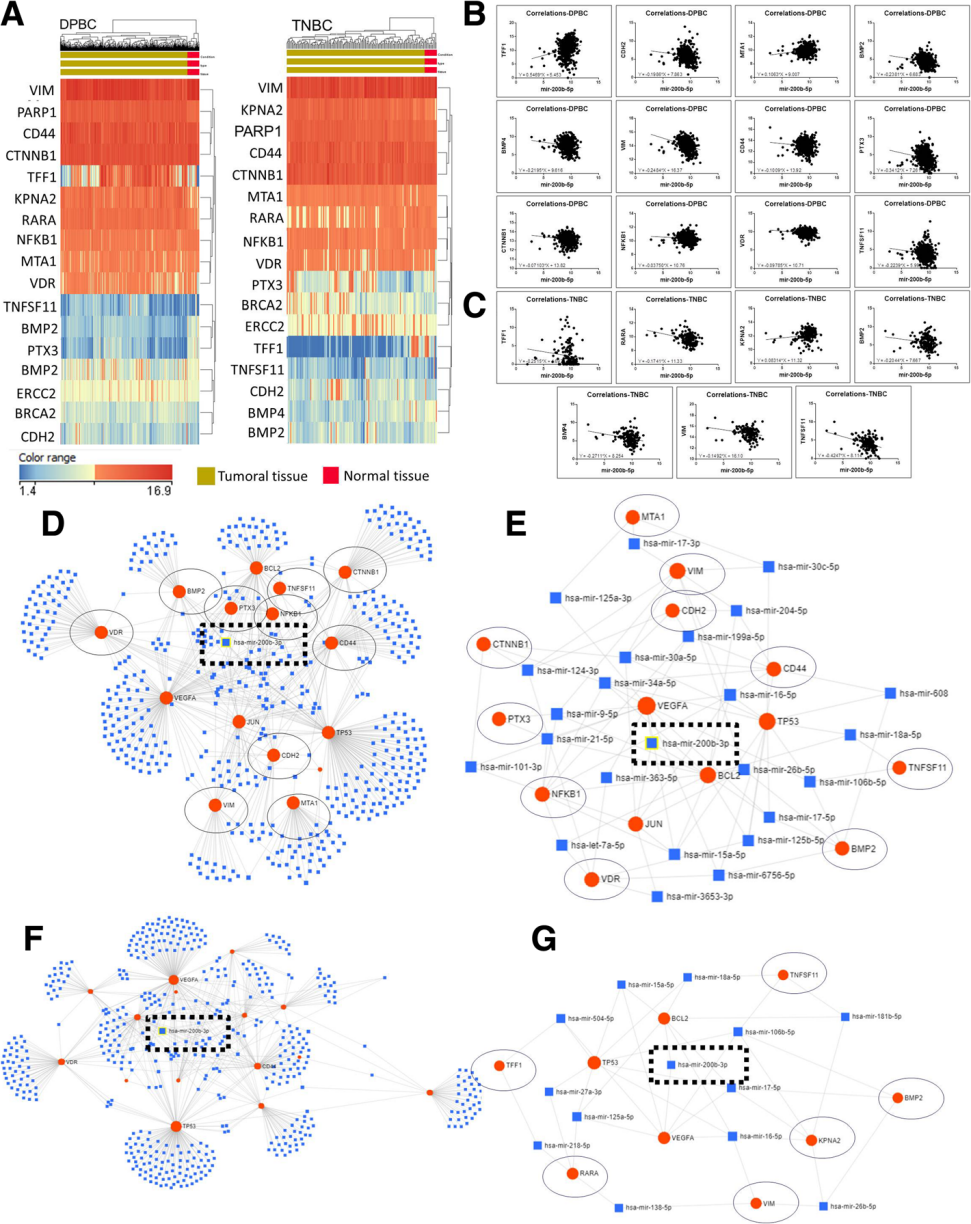
Correlation between miR-200b expression levels and the most relevant metastatic markers. (**a**) Heatmap for the metastatic markers in DPBC and TNBC breast cancer, based on TCGA data; (**b**) and (**c**) statistically significant correlation of microRNA-200b expression with the expression of the most relevant metastatic genes in DPBC and TNBC cancer (**d**) summary of metastatic responsive genes negatively or positively correlated with the expression level for miR-200b (**e**) direct and indirect interconnection of miR-200b with metastatic markers

Fig. 6a presents the heatmap for the metastasis-related genes in DPBC and TNBC. The correlation between miR-200b and these genes is illustrated in Fig. 6b for DPBC and in Fig. 6c for TNBC.

For the DPBC group, we found a statistically significant correlation between miR-200b and 12 genes. A positive correlation was observed for TFF1 and MTA1. A negative correlation was found for CDH2, BMP2, BMP4, VIM, CD44, PTX3, TNSF11, CTNNB1, NFKB1 and VDR. In the case of the TNBC group, miR-200b was positively correlated with KPNA2 and negatively correlated with TFF1, RARA, BMP2, BMP4, VIM and TNSF11. These data are summarized in Table 6.

**Table 6:** Metastatic genes correlated with miR-200b expression gene in DPBC and TNBC TCGA patient

Metastatic site	Gene	DPBC			TNBC		
		Pearson R	95% confidence interval	*P* value	Pearson R	95% confidence interval	P value
Brain metastasis marker	BRCA2	−0,02537	−0,1161 to 0,06580	0,5745	0,08805	−0,07530 to 0,2468	0,2760
	PARP1	0,02377	−0,06739 to 0,1145	0,5988	0,1341	−0,02884 to 0,2900	0,0963
Lung metastasis marker	TFF1	0,2018	0,1129 to 0,2876	0,0001	−0,1995	−0,3504 to −0,03850	0,0128
	RARA	0,02082	−0,07033 to 0,1116	0,6450	−0,3054	−0,4456 to −0,1506	0,0001
Liver metastasis	CDH2	−0,1995	−0,2854 to −0,1105	0,0001	−0,07199	−0,2316 to 0,09134	0,3734
	ERCC2	0,05412	−0,03709 to 0,1444	0,2308	0,003711	−0,1587 to 0,1659	0,9634
Bone metastasis	MTA1	0,2114	0,1227 to 0,2967	0,0001	0,1552	−0,007210 to 0,3097	0,0538
	KPNA2	−0,006807	−0,09777 to 0,08427	0,8803	0,1641	0,001867 to 0,3179	0,0413
	BMP2	−0,2626	−0,3454 to −0,1758	0,0001	−0,2030	−0,3536 to −0,04209	0,0113
	BMP4	−0,1170	−0,2058 to − 0,02625	0,0094	− 0,2244	− 0,3730 to − 0,06443	0,0050
	VIM	-0,3409	-0,4189 to − 0,2579	0,0001	-0,2438	-0,3906 to −0,08490	0,0022
	CD44	-0,09716	-0,1865 to −0,006196	0,0312	0,02089	-0,1419 to 0,1825	0,7964
	PTX3	-0,2637	-0,3464 to −0,1770	0,0001	-0,08625	-0,2451 to 0,07709	0,2859
	TNFSF11	-0,1752	-0,2620 to −0,08554	0,0001	-0,2297	-0,3779 to −0,07002	0,0040
	CTNNB1	-0,1828	-0,2694 to −0,09335	0,0001	-0,1193	-0,2762 to 0,04384	0,1394
	NFKB1	-0,1346	-0,2229 to −0,04411	0,0028	-0,1166	-0,2737 to 0,04652	0,1484
	VDR	-0,1421	-0,2301 to −0,05171	0,0016	-0,1324	-0,2885 to 0,03051	0,1005

## Discussion

Despite the late transition from pan-genomics to the post-genomics era, BC still remains one of the main causes of cancer related deaths [30]. TNBC is the most aggressive subtype of BC and it presents the worse clinical outcome among BC cases [2]. As follows, there is undeniable need for the development of novel diagnostic/prognostic markers that may also constitute therapeutic targets. Over the last few years, different research teams have explored the variation of miRNA profiles in relation to its diagnostic or prognostic potential [11, 21, 24, 31–33].

Certain miRNAs have a distinct expression profile specific for each BC subtype, which could prove to be a valuable diagnostic/prognostic tool. The bioinformatic analysis of the TCGA dataset is a powerful approach for characterizing miRNA expression patterns in large patients cohorts [27]. This allowed us to perform a comparison between tissue and circulating miRNAs. A partial correlation with the literature data was observed, especially in the case of miR-200 family members. This correlation was confirmed in both tissue and plasma samples. Specific patterns of plasma miRNAs appear to have distinct roles in metastasis. Furthermore, they can be related to the EMT, to invasion, or to late metastatic events, such as the establishment of metastatic tumors. However, different miRNA profiling studies failed to reach a consensus regarding the local versus systemic levels.

The miR-200 family members are regarded as the main regulators of EMT, invasion and metastasis. Moreover, it was recently discovered that miR-200 s contribute to the angiogenic process by targeting VEGF-A and its receptors [34, 35]. The inhibition of TGFβ receptor restores the normal ZEB/miR-200 balance and it leads to the overexpression of E-cadherin, resulting in reduced tumor dissemination [36]. As follows, miR-200 family is considered an early biomarker of metastasis [37, 38]. Our data supports this role of miR-200 as a general prognostic tool and a specific biomarker of early metastasis. This miRNA can be considered as a single evaluation tool or it can be correlated with the expression level of other coding or non-coding transcripts. Additionally, these other transcripts may function as direct or indirect targets, which can be seen in Fig. 6e.

The EMT process is considered as an efficient strategy adopted by epithelial cancer cells to promote local invasion and dissemination to distant organs [29]. This is supported by our evaluation of the miR-200 as an important metastatic marker, with a particular correlation in lung metastasis. The TFF1 gene was negatively correlated with the expression level for miR-200b in both breast cancer subtypes, meanwhile RARA gene was negatively correlated only in TNBC. We integrated these metastasis associated genes in a complex regulatory network. This could prove to be a useful tool for further experiments studying the mechanism of their action or the way they affect the clinical therapeutic outcomes in these Her-2- BC subtypes (Fig. 6e).

MiR-130 overexpression in breast cancer is related to EMT, invasion and metastasis. In addition**,** this microRNA is also connected with the downregulation of miR-200 [39, 40]. MiR-130 is known to have an active role in angiogenesis by modulating the expression of VEGF [41]. Another stand-out was miR-22, associated with poor clinical outcomes and the silencing of the TET-miR-200 axis in human breast cancer patients [42]. This microRNA was found to be specific for TNBC, when compared with DPBC.

The miR-29 family members were downregulated in various types of cancers and have been recognized mainly due to their tumor suppressive roles [43]. Lately, these molecules are presented as possible new biomarkers or therapeutic targets in BC, but with no direct implications in the TNBC pathogenesis [44, 45]. What’s more, the altered plasma levels of miR-29c and miR-200 were suggested to promote brain metastasis [46]. However, our results showed no correlation between the miR-200 expression level and the evaluated brain metastasis markers (BRCA1 and PARP1).

The miR-210 is another microRNA considered to have an effect over the clinical outcome of cancer patients [47]. The overexpression of this microRNA is correlated with a higher proliferation rate of the cancer cells. For BC patients, it was associated with an unfavorable prognostic [48], especially for Tamoxifen-treated patients [49]. The miR-210 up-regulation was observed specifically in patients with unresected tumours, lymph node involvement and metastases [50]. Some studies have established a correlation between miR-210 and the therapeutic response to Trastuzumab [50]. The miR-210 expression in TNBC was significantly higher than in DPBC [51]. A meta-analysis revealed that the increased level of miR-210 was related with a reduced overall survival [52]. In our study, the overlap analysis based on the TCGA data confirmed the results from previous studies. The miR-210 expression levels are similar in the plasma as well as the tumor tissue in both TNBC and DPBC.

In order to provide a more comprehensive overview of the interaction established between miRNA and mRNA, we constructed an IPA network. This is a helpful step towards a better understanding of the carcinogenic mechanisms as well as affected cellular pathways in TNBC and DPBC. As it was previously mentioned, EMT is an essential step in the metastatic cascade, because it leads to the activation of invasion and migration (Fig. 4d). Our study revealed a panel of miRNAs related to EMT that could become non-invasive biomarkers.

In this study, further details were revealed regarding the molecular basis of miR-200b involvement in BC metastasis, which can become a future clinical tool for establishing a more accurate prognostic. Our results demonstrated the increased sensibility of combined miRNA signature or miRNA-gene interaction.

The process of implementing a miRNA-based biomarker remains a challenge, the main problem being represented by the small patient cohort and the lack of a standardized method for evaluation. In addition, we need to take into account some of the patient characteristics such as dietary habits, environmental exposure, immune status and age. In this context, one miRNA with an altered expression level does not automatically have an oncogenic or a tumor suppressive role.

## Conclusion

We identified an aberrant miRNA expression pattern in the plasma of TNBC and DPBC patients. Our investigation found several miRNAs deregulated in the plasma of these patients, most of them being common for the HER2- subtypes of breast cancer. The miRNA specific signature for TNBC versus DPBC includes the downregulation of four miRNAs belonging to the miR-17-92 cluster (miR-17-5p, miR-20a, miR-20b, and miR-93), along with other miRNAs, such as miR-130, miR-22 and miR-29a/c. The overlap of circulating plasma and tissue miRNAs emphasizes the important role of miR-200b/c, miR-210 and miR-29c in TNBC tumorigenesis.

The regulatory mechanisms in cancer are more complex than one simple biomarker; miR-200b is a key element for the future answers given to the breast cancer mystery, especially considering that this microRNA is integrated in a regulatory network which acts in conjunction. As follows, not a single node, but the whole network affects the patient prognosis and response to therapy.

Nevertheless, the fluctuating levels of miR-200b provide a deep understanding over some of the mechanisms which drive the metastatic spread from the primary tumour. Controlling these EMT transcripts may increase the survival rate of the TNBC patients, due to their link with metastatic markers that promote cell adhesion, migration, and motility.

Further studies on a larger cohort of patients are needed to validate our findings. Also, much remains to be learned about the application of miRNA-based evaluation of treatment response and the early detection of recurrences.

## Additional files

Additional file 1: **Table S1.** TCGA tissue microRNAs differentially expressed for TNBC versus normal tissue (fold change ≤ -1.5 or ≥ 1.5, *p*-value < 0.05). (XLSX 38 kb)

Additional file 2: **Table S2.** TCGA tissue microRNAs differentially expressed for DPBC versus normal tissue (fold change ≤ -1.5 or ≥ 1.5, *p*-value < 0.05). (XLSX 38 kb)

Additional file 3: **Table S3.** TCGA tissue microRNAs differentially expressed for TNBC versus DPBC cancers (fold change ≤ -1.5 or ≥ 1.5, *p*-value < 0.05). (XLSX 34 kb)

Additional file 4: **Figure S1.** Heatmap for TNBC data from the TCGA data set. (TIFF 424 kb)

Additional file 5: **Figure S2.** Heatmap for DPBC data from the TCGA data set. (TIFF 419 kb)

Additional file 6: **Figure S3.** Heatmap for TNBC versus DPBC data from the TCGA data set. (TIFF 381 kb)

Additional file 7: **Figure S4.** Heatmap for plasma miRNA pattern for TNBC, DPBC and healthy controls. (PNG 14 kb)

## Abbreviations

AUC: Area under the curve
BC: Breast cancer
cDNA: Complementary DNA
DPBC: Double positive breast cancer patients
EMT: Epithelial to mesenchymal transition
ER: Estrogen receptor
IPA: Ingenuity Pathway Analysis
miRNAs: microRNAs
PR: Progesterone receptor
ROC: Receiving operator curves
TCGA: *The Cancer Genome Atlas*
TNBC: Triple negative breast cancer patients
