# Supplementary figures and images for: Aberrant miRNAs expressed in HER-2 negative breast cancers patient
Source: J Exp Clin Cancer Res. 2018 Oct 20;37:257. doi: 10.1186/s13046-018-0920-2 (PMC6196003; doi:10.1186/s13046-018-0920-2)

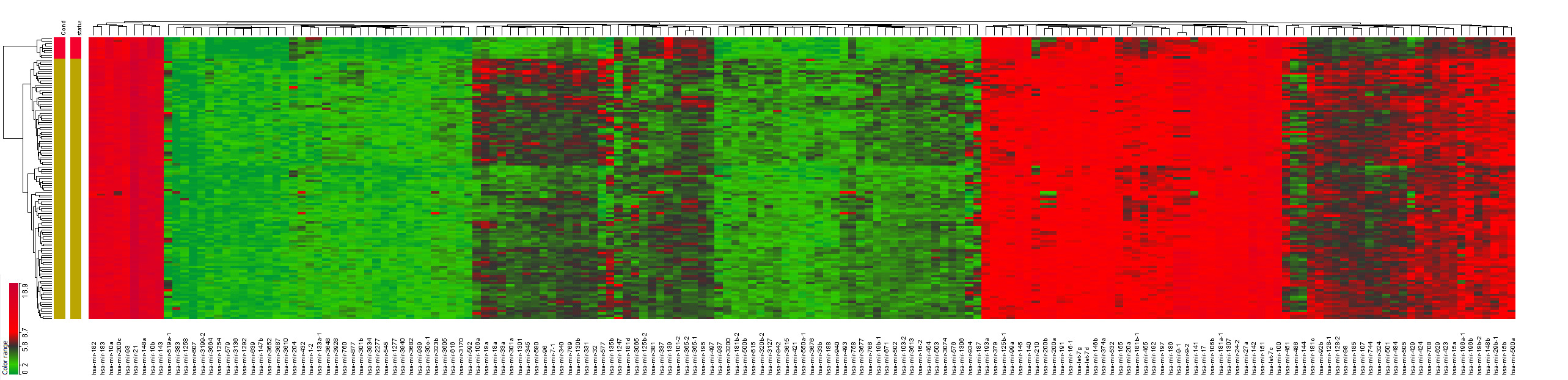

Supplement: Supplementary file 4 — Figure S1. Heatmap for TNBC data from the TCGA data set. (TIFF 424 kb) [file 13046_2018_920_MOESM4_ESM.tiff]

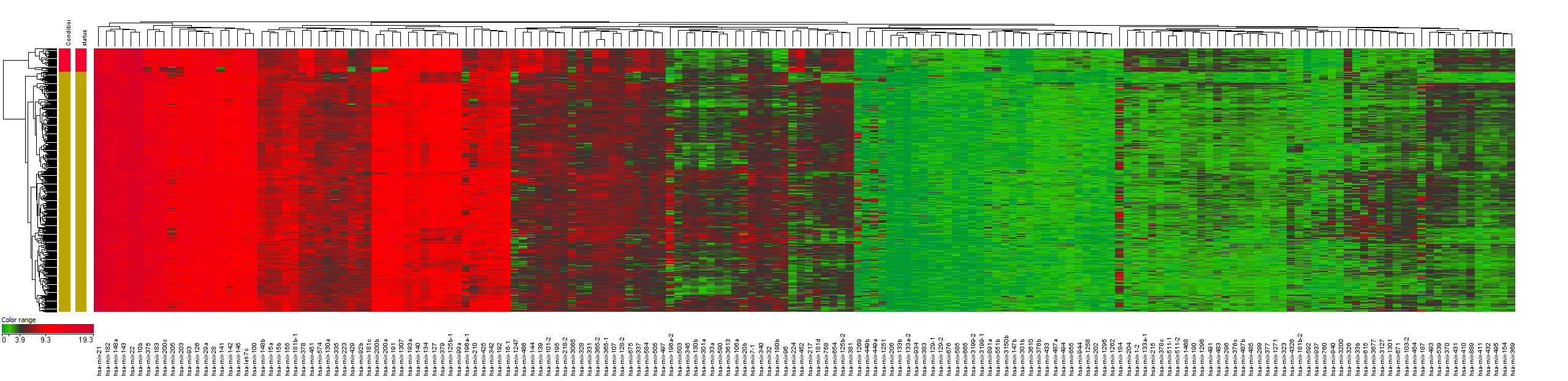

Supplement: Supplementary file 5 — Figure S2. Heatmap for DPBC data from the TCGA data set. (TIFF 419 kb) [file 13046_2018_920_MOESM5_ESM.tiff]

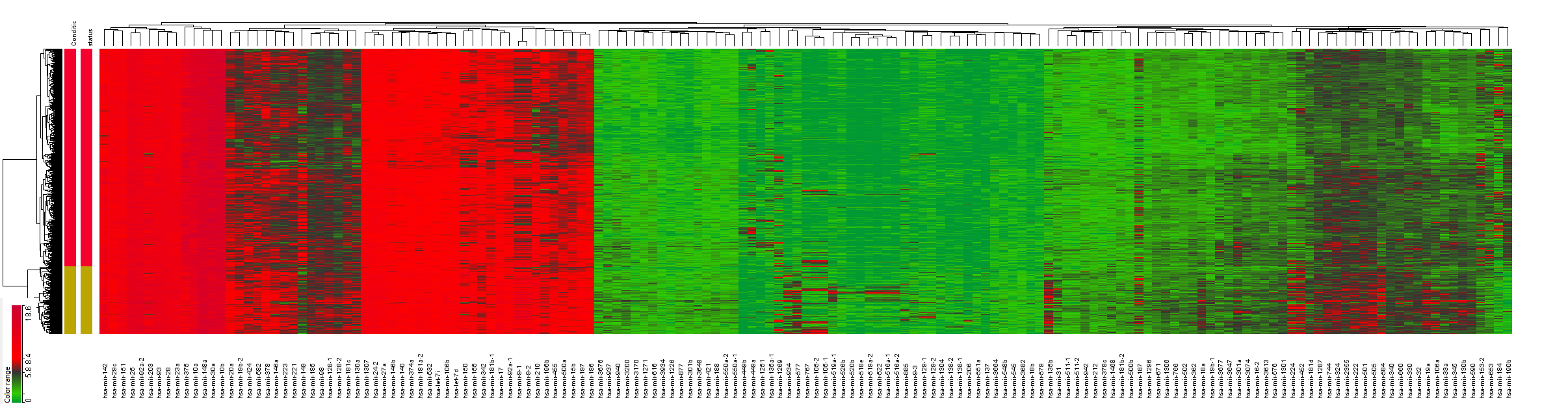

Supplement: Supplementary file 6 — Figure S3. Heatmap for TNBC versus DPBC data from the TCGA data set. (TIFF 381 kb) [file 13046_2018_920_MOESM6_ESM.tiff]

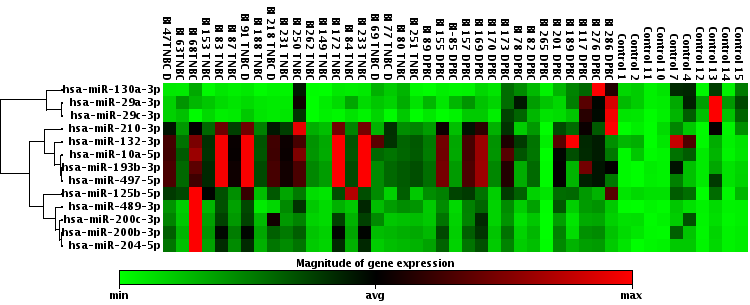

Supplement: Supplementary file 7 — Figure S4. Heatmap for plasma miRNA pattern for TNBC, DPBC and healthy controls. (PNG 14 kb) [file 13046_2018_920_MOESM7_ESM.png]
